# Platelet-Rich Plasma Obtained with Different Anticoagulants and Their Effect on Platelet Numbers and Mesenchymal Stromal Cells Behavior In Vitro

**DOI:** 10.1155/2016/7414036

**Published:** 2016-06-02

**Authors:** Ronaldo José Farias Corrêa do Amaral, Nemias Pereira da Silva, Natália Ferreira Haddad, Luana Siqueira Lopes, Fábio Dias Ferreira, Ricardo Bastos Filho, Paola Alejandra Cappelletti, Wallace de Mello, Eric Cordeiro-Spinetti, Alex Balduino

**Affiliations:** ^1^Excellion Serviços Biomédicos, Amil/UnitedHealth Group, 25651-000 Petrópolis, RJ, Brazil; ^2^Laboratório de Biologia e Tecnologia Celular, Universidade Veiga de Almeida, 20270-150 Rio de Janeiro, RJ, Brazil; ^3^Universidade Federal Fluminense, 24033-900 Niterói, RJ, Brazil; ^4^Centro Universitário Celso Lisboa, 20950-091 Rio de Janeiro, RJ, Brazil

## Abstract

There are promising results in the use of platelet-rich plasma (PRP) for musculoskeletal tissue repair. However, the variability in the methodology for its obtaining may cause different and opposing findings in the literature. Particularly, the choice of the anticoagulant is the first definition to be made. In this work, blood was collected with sodium citrate (SC), ethylenediaminetetraacetic acid (EDTA), or anticoagulant citrate dextrose (ACD) solution A, as anticoagulants, prior to PRP obtaining. Hematological analysis and growth factors release quantification were performed, and the effects on mesenchymal stromal cell (MSC) culture, such as cytotoxicity and cell proliferation (evaluated by MTT method) and gene expression, were evaluated. The use of EDTA resulted in higher platelet yield in whole blood; however, it induced an increase in the mean platelet volume (MPV) following the blood centrifugation steps for PRP obtaining. The use of SC and ACD resulted in higher induction of MSC proliferation. On the other hand, PRP obtained in SC presented the higher platelet recovery after the blood first centrifugation step and a minimal change in MSC gene expression. Therefore, we suggest the use of SC as the anticoagulant for PRP obtaining.

## 1. Introduction

Platelet-rich plasma (PRP) is a blood-derived product in which platelets are concentrated at least five times in plasma above the baseline of that in the whole blood [1]. PRP is being investigated as an autologous product to improve tissue repair in different conditions and lesions, especially for musculoskeletal tissues, such as chondral lesions [2–4], tendinopathies [5–7], muscle strains [8, 9], and bone repair [10, 11]. Besides its clinical application, PRP may be an efficient substitute to fetal bovine serum in cell culture [12–15]. Its therapeutic potential is based mainly on the growth factors present in platelet's alpha granules [16], such as transforming growth factor beta (TGF-*β*) [17], vascular endothelial growth factor (VEGF) [18], and platelet-derived growth factor (PDGF) [19], which have already been demonstrated to play important roles in tissue repair. When platelets are concentrated and activated, it is expected that the concentration of the factors released reaches three to five times of that found in the plasma [16].

The general methodology to obtain PRP involves the collection of whole blood with anticoagulants, followed by one or two centrifugation steps. After a first low-speed centrifugation, erythrocyte-free platelet concentrated plasma is recovered and submitted to high-speed centrifugation. Platelet-poor plasma is then discarded and the remaining platelet pellet is homogenized into what is regarded as PRP [16]. Several aspects on this method are still under debate, such as number of centrifugations, presence of leukocytes, and use and type of platelet activator and anticoagulants [20]. When no anticoagulant is used, a blood clot will form, and serum can be obtained but without increase in platelet concentration [21]. In the case of PRP obtaining, coagulation is not intended to occur prior to platelet concentration; hence, blood must be collected in the presence of anticoagulants.

For transfusion purposes, blood is usually collected in bags containing citrate phosphate dextrose adenine (CPDA-1) solution, as anticoagulant [22, 23], from which a platelet concentrate is obtained by double centrifugation of the whole blood or apheresis. Platelets concentrates obtained by such methodologies may also be used for promoting tissue repair [24]. On the other hand, recent PRP formulations for autologous applications are usually prepared in collection tubes containing citrate solutions, in the form of sodium citrate [25–29] or ACD-A [30–32]. This last, ACD, is present in the majority of available commercial kits for PRP production [33, 34]. In other cases, heparin [35, 36] or EDTA [37] can be used. For clinical investigations, EDTA is commonly used in hematology tests, SC in coagulation tests, and ACD in plasma levels measurement of platelet-derived components [38]. Therefore, our goal was to analyze how the choice of anticoagulant for blood collection would modulate PRP characteristics as well as its effects on mesenchymal stromal cell culture.

## 2. Materials and Methods

### 2.1. Ethics Statement

All of the experimental procedures were approved by the Ethics Research Committee of the Pró-Cardíaco Hospital, Rio de Janeiro (CAAE: 14878813.4.0000.5533), and all donors signed an informed consent.

### 2.2. PRP Obtaining

PRP was obtained as previously described, with minor modifications [39]. Peripheral blood was collected from nine volunteer donors (6 men and 3 women) using blood collection tubes containing sodium citrate (SC) (Vacutainer®, Ref: 369714; BD Biosciences, San Jose, CA), ethylenediaminetetraacetic acid (EDTA) (Vacutainer, Ref: 367861; BD Biosciences), or anticoagulant citrate dextrose (ACD) solution A (Vacutainer, Ref: 364606; BD Biosciences) solution. The blood collected in one ACD tube was maintained in the same tube or divided into three polypropylene tubes containing no anticoagulant (Falcon*™*, Ref: 352063; BD Biosciences), termed as ACD-2.

Tubes were centrifuged at 300 g for 5 minutes (Megafuge® 40, Thermo Fisher Scientific, Waltham, MA). Supernatant containing plasma and platelets, termed as platelet-rich plasma 1 (PRP1), was collected from each tube and transferred to new polypropylene tubes containing no anticoagulant. In the case of ACD and ACD-2, after platelet counting, PRP1 from the same donors was mixed for the next experiments. Then, PRP1 was centrifuged at 700 g for 17 minutes. Supernatant was collected, namely, platelet-poor plasma (PPP). Part of the PPP from each tube was used to resuspend the platelet pellet, forming the platelet-rich plasma 2 (PRP2), in order to achieve the expected concentration of 10^6^ platelets/*µ*L. The platelets in PRP2 were activated by adding 1 M CaCl_2_ (final concentration of 20 mM) and incubated at 37°C for 1 hour. After clot formation, tubes were maintained at 4°C during 16 hours to allow clot contraction. Finally, tubes were centrifuged at 3000 g for 20 minutes and the supernatant was collected, termed as platelet-rich plasma releasate (PRPr). The PRPr was freezed at −80°C until thawing for experimental use.

### 2.3. Hematological Analysis

Counting of platelets, red blood cells, white blood cells, and analysis of mean platelet volume (MPV) were determined in whole blood, PRP1, PRP2, and PPP fractions. Those analyses were performed with a hematological analyzer (Mindray BC 2800, Perdizes, SP, Brazil). Platelet recovery after the first centrifugation step, expressed as a percentage, was calculated by dividing the total number of platelets in PRP1 by the total number of platelets in whole blood.

### 2.4. Quantification of Growth Factors

PRPr-derived TGF-*β*1 and VEGF were quantified using ELISA kits (Ref: KAC1688 and Ref: KHG0111; Invitrogen*™*, Thermo Fisher Scientific) according to manufacturer's instructions. Absorbance was determined using a microplate reader (Multiskan GO, Thermo Fisher Scientific).

### 2.5. Bone Marrow-Derived Mesenchymal Stromal Cell (BM-MSC) Isolation and Culture

120 mL of bone marrow was obtained after donation with informed consent from two donors (a 41-year-old woman, whose cells were used for cell viability assays, and a 60-year-old man, whose cells were used for gene expression analysis). Mononuclear cells were separated using the Sepax system (Biosafe, Eysins, Switzerland), according to manufacturer's instructions, and plated at 4 × 10^5^ cells/cm^2^ in Minimum Essential Medium Eagle Alpha Modification (alpha MEM) (Cultilab, Campinas, SP, Brazil) supplemented with 10% fetal bovine serum (FBS) (Gibco) in T 150 cm^2^ culture flasks (Corning Incorporated, Corning, NY) and maintained in a 5% CO_2_ incubator at 37°C. After 5 days, medium was changed and nonadherent cells were discharged. Medium was changed every two days. This was termed as “primary culture.” After approximately 10 days, 70–80% confluence, cells were detached from culture flasks using 0.05% trypsin solution (Gibco®, Thermo Fisher Scientific) and replated onto new culture flasks at a density of 8 × 10^3^ cells/cm^2^. After first trypsinization, culture was termed as at “passage #1.” Experiments were performed until “passage #5.”

### 2.6. Cell Viability Assay

The analysis of cell viability was performed by incorporation with thiazolyl blue tetrazolium bromide (MTT assay) (Sigma Aldrich, São Paulo, SP, Brazil). Cells (passage #3) were plated at a density of 5 × 10^3^ cells/cm^2^ in duplicate in 48-well plates (Corning Incorporated) in alpha MEM (Cultilab) supplemented with 10% FBS (Gibco), 1% PRPr, 2.5% PRPr, or 5% PRPr. Four different PRPr donors were used. In another group, the four samples were pooled with equal proportions of each donor, namely, PRPr MIX. After 8 days of culture, 0.5 mg/mL MTT was added. Medium was removed after 4 hours of incubation, and 400 *µ*L/well of DMSO was added to dissolve the reduced formazan product. The volume in each of the 48 wells was split into two wells in a 96-well plate (Corning Incorporated). Finally, the plate was read in a microplate reader (Multiskan GO, Thermo Fisher Scientific) at 570 nm. Cell culture medium was not changed during this experiment.

### 2.7. Gene Expression Evaluation

Cells (passage #5) were cultured in alpha MEM (Cultilab) supplemented with 10% FBS (Gibco), 1% PRPr, 2.5% PRPr, or 5% PRPr. The PRPr was used as a pool of four different donors. After five days of culture, total RNA was extracted using TRIzol® (Ambion®, Thermo Fisher Scientific). RNA concentration was determined using a Nanodrop 2000 UV-Vis spectrophotometer (Thermo Fisher Scientific) and 2 *µ*g was reverse-transcripted into complementary DNA (cDNA) using SuperScript® First-Strand Synthesis System for RT-PCR (Invitrogen, #11904-018) in a total reaction volume of 20 *µ*L, following manufacturer's protocol. Oligonucleotides and probes for qPCR were purchased from Applied Biosystems (TaqMan Gene Expression Assay, #4331182): HPRT1 (Hs02800695_m1), which was analyzed as the housekeeping gene, SOX9 (Hs00165814_m1), RUNX2 (Hs00231692_m1), PPARG (Hs01115513_m1), and POU5F1 (Oct-4) (Hs0099634_9H). qPCR reactions were performed in an Applied Biosystems 7500 Standard Time PCR System in a 20 *µ*L reaction volume using TaqMan® Universal Master Mix II, with UNG (Applied Biosystems, #4440038), according to manufacturer's instructions. Analysis was performed using the ΔΔCt method [40].

### 2.8. Statistical Analysis

Data were analyzed using a two-tailed paired *t*-test for the hematological analysis, where a group of the same donors were analyzed with different anticoagulants. In the case of growth factor quantification and cell culture experiments, where pairing of samples did not necessarily occur, a two-tailed unpaired *t*-test was performed. Statistical significance was considered when *p* < 0.05.

## 3. Results

### 3.1. Effect of Different Anticoagulants on Initial Platelet Counting and Recovery

**Figure 1:**
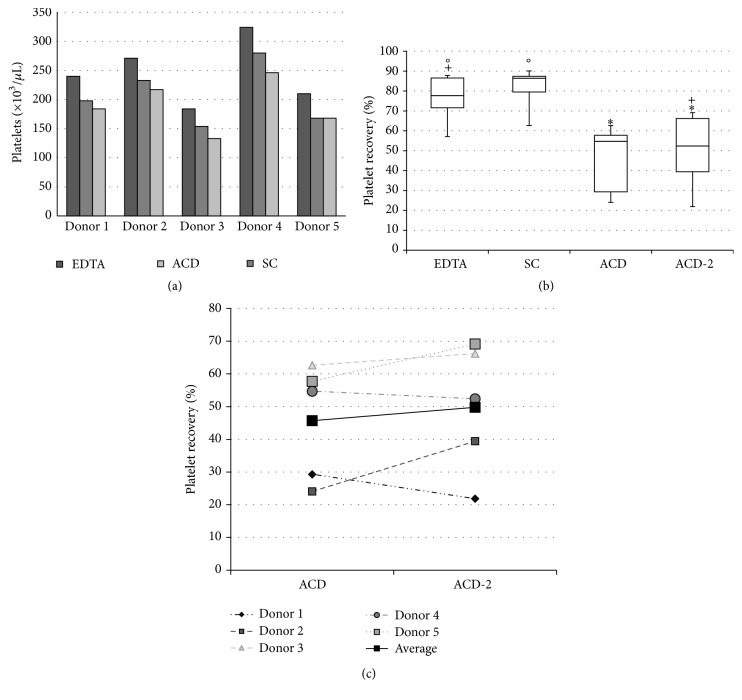
Platelet yield and recovery in blood collected with different anticoagulants. Blood was collected in EDTA, SC, and ACD in five different donors and platelet concentration was quantified (a) as well as platelet recovery after the first centrifugation step (b). An individual analysis between ACD and ACD-2 of platelet recovery was also performed (c). Data are expressed as bar (a), box (b), and dot (c) plots. Similar symbols in (b) correspond to statistic similarity among groups (*p* > 0.05).

Blood samples were collected from five different donors in tubes containing EDTA, SC, or ACD, and platelets were counted in an automated system. Blood samples collected with EDTA yielded higher numbers of platelets, followed by SC and ACD (Figure 1(a)). In average, platelet counting in SC was 16.28% lower than that in EDTA, while that in ACD was 23.01% lower than in EDTA and 7.94% lower than in SC. However, platelet recovery, regarding the total number of platelets obtained after the first centrifugation step, was higher in the presence of SC compared to EDTA and ACD. The average of platelet recovery in EDTA and SC was 76.15% and 81.21%, respectively. Strikingly, platelet recovery in samples collected with ACD was 45.71%, almost half of those when using EDTA or SC. All three anticoagulants tested herein were purchased in commercially distributed tubes. ACD containing tube was bigger—taller and larger—compared to EDTA and SC.

In order to verify if the lower platelet recovery was related to the tube format, PRP was obtained from blood samples anticoagulated in ACD using tubes of similar size compared to EDTA and SC (ACD-2). Platelet recovery improved (49.82%) but remained much lower than those recovered when using EDTA (76.15%) and SC (81.21%). Values from ACD-2 were statistically different from those obtained using SC (*p* < 0.05) but not when compared to EDTA (*p* > 0.05) (Figure 1(b)). If analyzed separately, it was possible to observe that platelet recovery has increased in three of the five donors when using ACD-2 instead of ACD, especially in donor 2, with an increase of 63.74%, while it has decreased in two donors, especially in donor 1, with a decrease of 25.48% after the distribution of blood into the smaller tubes (Figure 1(c)). In average, platelet concentration in PRP2 was 1,009 ± 57 × 10^3^/*µ*L in EDTA samples, 582 ± 108 × 10^3^/*µ*L in SC samples, 726 ± 200 × 10^3^/*µ*L in ACD samples, and 664 ± 170 × 10^3^/*µ*L in ACD-2 samples. All values were statistically similar between each other (*p* > 0.05), except between EDTA and SC (*p* < 0.05) (data not shown).

**Figure 2:**
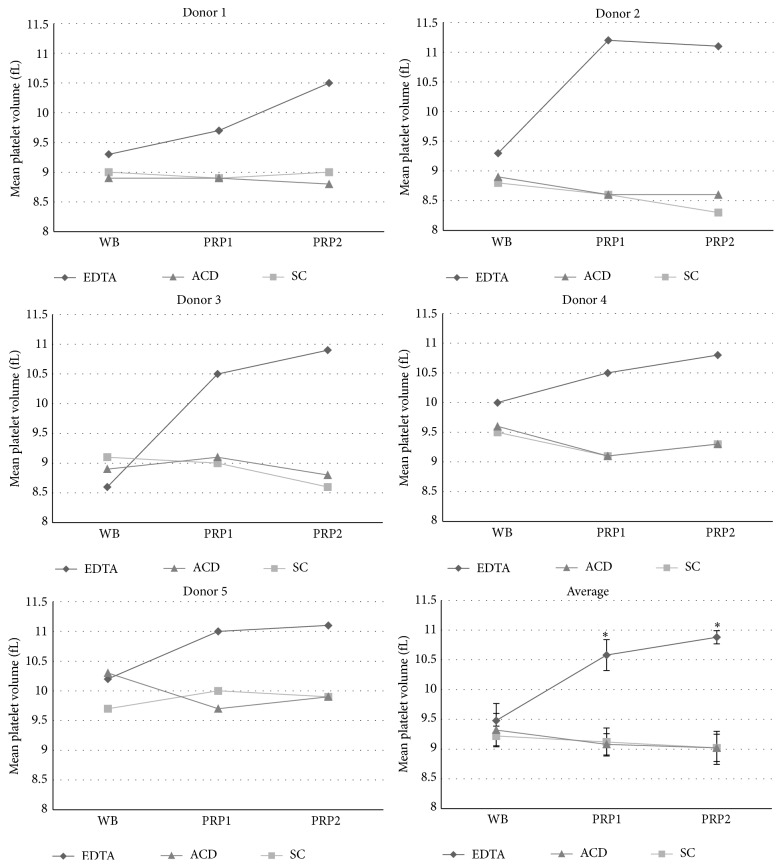
Mean platelet value quantification of samples containing different anticoagulants. Mean platelet value was quantified in five different donors in whole blood (WB), PRP1, and PRP2, in tubes containing EDTA, SC, or ACD solution. The average values of the five different donors are also represented in the figure. “*∗*” corresponds to statistical difference between EDTA and SC groups as well as EDTA and ACD groups (*p* < 0.05).

Although the mean platelet volume (MPV), which is related to platelet size and indicates its degree of activation, was similar when whole blood (WB) samples were anticoagulated in all three anticoagulants tested, it increased progressively following the two centrifugation steps in EDTA group in all donors (in average an increase of 11.60% after the first centrifugation step and an additional increase of 2.84% after the second centrifugation step, totaling 14.44% increase compared to whole blood). This was not observed when WB was anticoagulated in SC and ACD (Figure 2).

### 3.2. TGF-*β*1 and VEGF Release from Platelet-Rich Plasma in Different Anticoagulants

**Figure 3:**
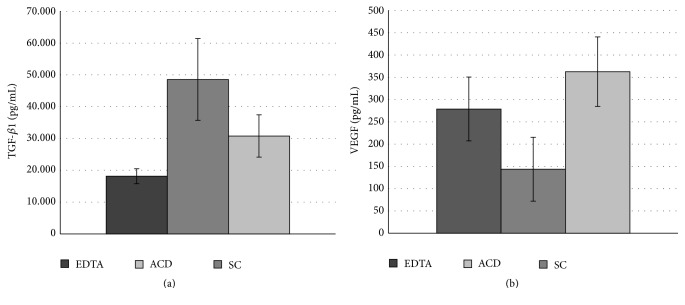
Growth factor quantification in PRPr obtained in different anticoagulant. TGF-*β*1 (a) and VEGF quantification (b). Data are expressed as mean, and error bars correspond to standard error.

Up to this point, it was clear that the anticoagulant has an impact on platelet recovery after blood centrifugation. However, we questioned if it would also change growth factors release from recovered platelets. For that, we quantified TGF-*β*1 and VEGF levels in an ELISA assay. Growth factors concentrations were similar between anticoagulant groups (*p* > 0.05) for both TGF-*β*1 and VEGF. TGF-*β*1 concentration was 18,146.99 ± 2,370.33 pg/mL in EDTA; 48,559.10 ± 12,839.86 pg/mL in SC; and 30,786.15 ± 6,654.49 pg/mL in ACD (Figure 3(a)). VEGF concentration was 278.88 ± 71.78 pg/mL in EDTA, 143.65 ± 71.63 pg/mL in SC, and 362.70 ± 77.95 pg/mL in ACD (Figure 3(b)).

### 3.3. Bone Marrow-Derived Mesenchymal Stromal Cell Culture

**Figure 4:**
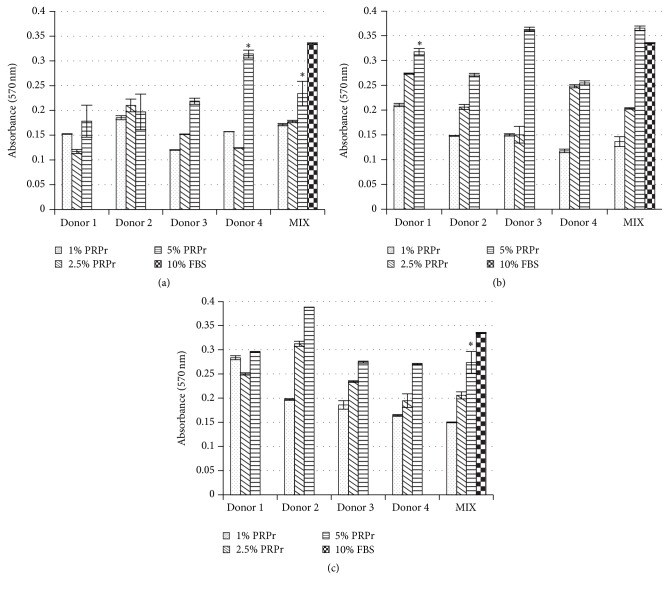
BM-MSC viability in PRPr obtained with different anticoagulant. Absorbance at 570nm was measured after MTT viability assay of cells cultivated in different PRPr concentrations, obtained with EDTA (a), SC (b), and ACD (c), as well as 10% FBS (control). Data are expressed as mean, and error bars correspond to standard error. “*∗*” corresponds to statistical similarity with 10% FBS (*p* > 0.05).

**Figure 5:**
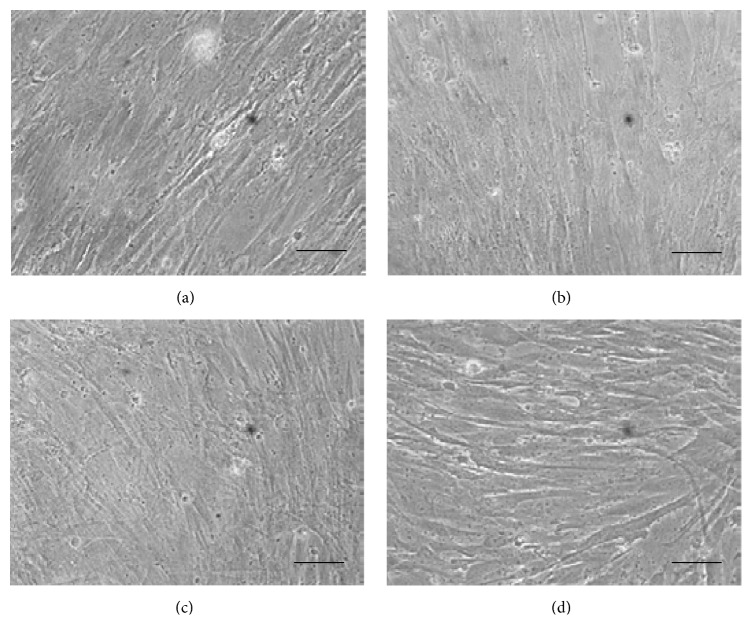
Photomicrography of BM-MSC. Cells cultivated for eight days in medium supplemented with 10% FBS (a) or a pool from four donors of 5% PRPr obtained from collection tubes containing EDTA (b), SC (c), or ACD (d). Phase contrast, 200x magnification, and scale bars: 50 *µ*m.

In order to show the effects of factors released from platelets obtained using different anticoagulants on modulating cell expansion in vitro, we used the MTT cell viability assay to analyze BM-MSC proliferation in the presence of different concentrations of PRPr. FBS-supplemented culture medium was used as reference (Figure 4). PRPrs were tested separately and mixed (MIX). All concentrations of PRPr tested from all donors were able to stimulate cell proliferation in vitro. As expected, the higher concentration tested (5%) stimulated the higher proliferative rate in vitro, regardless of the anticoagulant used. However, for this concentration, in average, cell proliferation was lower in the presence of EDTA derived PRPr compared to SC and ACD (Figure 4). In addition, cells maintained their fibroblast-like morphology regardless of the anticoagulant type (Figure 5).

### 3.4. Modulation of Bone Marrow-Derived Mesenchymal Stromal Cell Gene Expression by Platelet-Rich Plasma Culture

**Figure 6:**
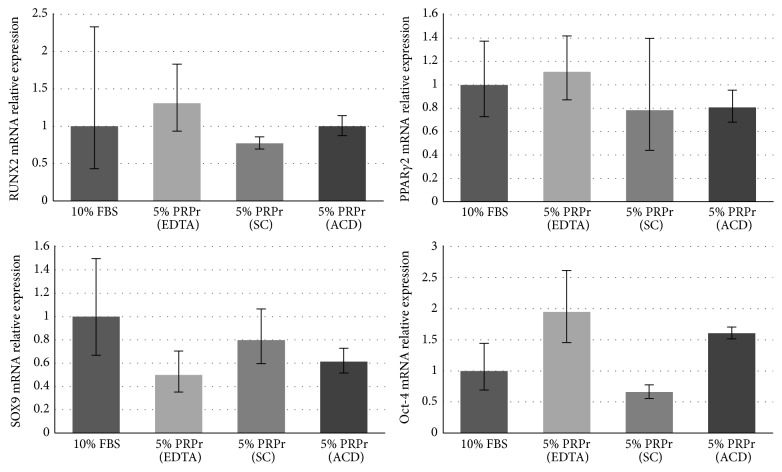
Relative gene expression. RUNX2, PPAR*γ*2, SOX9, and Oct-4 gene expression in cells cultured in medium supplemented with 10% FBS (control group) or 5% PRPr obtained with EDTA, SC, or ACD. Data are expressed as relative quantification of gene expression (RQ). Upper and lower error bars correspond to RQ maximum and RQ minimum, respectively.

We also analyzed gene expression of cells expanded in vitro (Figure 6). Only cells cultured in 5% PRPr were tested. Using 10% FBS as reference, RUNX2 was slightly upregulated in EDTA group and downregulated in SC group. PPAR*γ*2 was slightly upregulated in EDTA group and downregulated in SC and ACD groups. SOX9 was downregulated in all groups. Oct-4 was upregulated in EDTA and ACD groups and downregulated in SC group. Although, in general, the gene expression was similar between the PRPr groups, especially when observing the maximum and minimum relative quantification of gene expression, the SC group presented the smallest variation compared to the control group, by analyzing the average relative expression of the four genes in the PRPr groups compared to the FBS group. In average, SC relative gene expression was 24.73% different from the control group, while EDTA was 46.79% and ACD was 29.74% different.

## 4. Discussion

Platelet-rich plasma (PRP) is currently one of the main strategies to promote musculoskeletal tissues repair. There are several reports in the literature evidencing its potential in clinical trials [2–11] as well as in vitro analysis [12–15]. As a cost-effective source of autologous growth factors that can affect stem cells proliferation and differentiation, it is being increasingly investigated as a supplement, adjuvant, carrier, or scaffold for stem cells-based therapeutics [41–45]. However, the lack of standardization between the methodology to obtain and use PRP among different groups may hamper the development of this technology [20]. The use of anticoagulant to collect blood is a major issue. The present work aimed to verify PRP obtaining with three types of commercially available blood collection tubes containing EDTA, SC, or ACD as anticoagulants.

Platelet counting was higher in blood collected in tubes containing EDTA, followed by SC and ACD. Indeed, it has been previously shown that platelet count in EDTA can be higher than in citrate anticoagulants [46]. Moreover, when EDTA is added to citrated samples, it can enhance platelet count in whole blood [47]. Platelet recovery after the first centrifugation step was diminished in ACD tubes compared to EDTA and SC. Additionally, a higher concentration of PDGF-BB was found in PRP obtained with EDTA compared to ACD [48]. In our case, we tried to enhance platelet recovery in ACD tubes by dividing its content into smaller tubes (12 × 75 mm × 5 mL) with no additional anticoagulant. Although no statistical difference has been detected between those two ACD forms, the splitting of blood in the smaller tubes resulted in a similar platelet recovery compared to EDTA group. Since ACD tube is bigger (16 × 100 mm × 8.5 mL) than EDTA (13 × 75 mm × 4.0 mL) and SC tubes (13 × 75 mm × 4.5 mL), it is possible that the lower platelet recovery is due not only to the type of anticoagulant itself but also to the tube format. Particularly, the tube format may have superior influence on whole blood/plasma than serum centrifugation, in view of its higher viscosity [49]. For the following experiments, we decided to mix ACD and ACD-2 PRP1 from the same donors, since it is comprised of the same type of anticoagulant and since the following centrifugation to prepare PRP2 was performed in the same type of tube for all groups analyzed.

In order to verify if the centrifugation steps influenced platelets morphology, we quantified the mean platelet volume (MPV) in whole blood, PRP1, and PRP2 obtained with different anticoagulants. The MPV in the EDTA group, but not in SC and ACD groups, has increased after the centrifugation steps, which may be an indicator of platelet activation [50, 51]. Indeed, a higher MPV is expected in whole blood collected in EDTA compared to citrated samples [46]. In addition, EDTA may change platelet morphology from a discoid to an irregular spherical shape [52]. Indeed, the use of EDTA faces ethical issues, such as being pointed to as a persistent pollutant in natural environments [53], and impediments of use in certain countries [54]. ACD and citrate-theophylline-adenosine-dipyridamole (CTAD) are also more efficient in maintaining platelet morphology than heparin and SC. In that case, it has also been shown that PRP obtained with ACD and CTAD resulted in higher TGF*β*-1 concentration and induction of MSC proliferation [55]. In another previous study, EDTA, SC, and ACD were compared as maintainers of platelet responsiveness to aggregation inducers. ACD was the most capable to maintain intraplatelet signal transduction mechanisms during PRP formulation [56]. The same group, lately, showed that ACD was also capable of maintaining platelet functions for periods of time superior to SC [57]. In our case, we could not find any difference in the capability of platelet activation and clot formation in PRP2 obtained with the three different anticoagulants.

Our next step was to evaluate the effect of PRP obtaining in cell culture. For that, we normalized the platelet concentration to ideally 1000 × 10^3^/*µ*L in all groups, in a way that the results would not correlate to platelet concentration but to the type of the anticoagulant. This platelet concentration in PRP is being pointed to as a therapeutic concentration for in vivo purposes [1]. In bone, for example, lower concentrations are unsatisfactory and higher concentrations are inhibitory to promote tissue repair [11]. The only statistical difference observed in platelet concentration in PRP2 was between EDTA and SC group. The lower value in SC group may be attributed to a difficulty to resuspend platelet pellet. Platelet concentrate bags prepared with citrate samples, in form of ACD, contain more aggregates than bags prepared with EDTA [52]. Moreover, the effects of PRPr on cell culture were always compared to 10% FBS medium supplementation. In spite of being a xenogeneic serum with ethical and scientific issues [58], FBS is still widely used in cell culture [59] and MSC in vitro expansion [60].

In our study, we could not find differences in TGF*β*-1 and VEGF concentration among the anticoagulants. It could be possibly due to the similar platelet concentration between groups. In average, TGF-*β*1 concentration varied from 18.15 ng/mL in EDTA to 48.56 ng/mL in SC. Other literature reports present concentrations superior to our finding: 120 ng/mL [61], 169 ng/mL [62], and 89 ng/mL in a PRP with platelet concentration 4.69 times superior to whole blood baseline but 20 ng/mL in a PRP with platelet concentration 1.99 times superior to whole blood baseline [63]. VEGF concentration ranged from 143.65 pg/mL in SC to 362.70 pg/mL in ACD. Other reports present concentrations varying from 50 pg/mL [64] to 155 ng/mL [61]. Nevertheless, our results were superior to some commercially available kits [65].

As expected, PRPr induced cell proliferation. Although there were variations comparing the influence on cell proliferation between donors, possibly due to an inherent difference on growth factors and other molecules content among each platelet granule, an evident pattern emerged: the concentration of 5% PRPr in cell culture medium was sufficient to induce cell proliferation in a similar level to 10% FBS. Additionally, SC and ACD-derived PRPr presented greater effects over cell proliferation compared to EDTA group. Cell morphology was not changed among groups. BM-MSC maintained their fibroblastic morphology regardless of the anticoagulant. Although there are still controversies in the literature regarding PRP effects on BM-MSC differentiation, there is a consensus on its effect as an inducer of proliferation [66].

As a final analysis of PRPr effects on BM-MSC, we observed slight modulations in the expression of the master genes for the osteogenic (RUNX2), adipogenic (PPAR*γ*2), and chondrogenic (SOX9) lineages [67], as well as Oct-4, a gene related to maintenance of stemness [68, 69]. Particularly, SOX9 expression was downregulated. Indeed, there is a discussion in the literature regarding the PRP effects on chondrogenesis, with some groups claiming its inducing effects [70, 71] and others its inhibitory effects [72, 73]. Recently, our group has showed that it can be dependent on the concentration of PRPr used in cell medium, with lower concentrations inducing chondrogenesis and higher concentrations inhibiting it [74]. Oct-4 expression presented an intense variability, with upregulation in EDTA and ACD groups and downregulation in SC group. Whether this can be an indicator of the maintenance of cells stemness or not, it must be further investigated. In general, gene expression was similar between PRP groups, although SC was the group that presented the smaller variation compared to 10% FBS culture, evidencing that cells presented the lightest changes in their phenotype with this treatment.

## 5. Conclusion

The literature provides a variety of methodologies to obtain PRP. The first variation is the methodology to collect blood and the anticoagulant used. In this paper, we analyzed the effects of three different anticoagulants, obtained in commercially available tubes, on PRP obtaining. Although no significant change in the amount of growth factors released was observed, some features could be highlighted. The blood collection in tubes containing EDTA resulted in higher platelet yield in the whole blood. However, this was accompanied by an increase of MPV following the centrifugation steps, which is an indicator of change in platelet morphology. On the other hand, the use of tubes containing citrate solutions resulted in a greater induction of MSC proliferation. Particularly, the obtaining in SC resulted in the higher platelet recovery after the first centrifugation step. If ACD is used, the reduction of the tube size may increase platelet recovery. In addition, the PRP obtained in SC presented the smallest variation in MSC gene expression compared to cells cultured in the presence of 10% FBS. Therefore, in order to obtain a bigger amount of platelets and induce MSC proliferation without dramatically interfering with their phenotype, we suggest the use of SC as anticoagulant for PRP acquisition.

## References

[1] Marx R. E. (2001). Platelet-rich plasma (PRP): what is PRP and what is not PRP?. *Implant Dentistry*.

[2] Filardo G., Kon E., Buda R. (2011). Platelet-rich plasma intra-articular knee injections for the treatment of degenerative cartilage lesions and osteoarthritis. *Knee Surgery, Sports Traumatology, Arthroscopy*.

[3] Sánchez M., Azofra J., Anitua E. (2003). Plasma rich in growth factors to treat an articular cartilage avulsion: a case report. *Medicine and Science in Sports and Exercise*.

[4] Kon E., Filardo G., Di Matteo B., Marcacci M. (2013). PRP for the treatment of cartilage pathology. *The Open Orthopaedics Journal*.

[5] Taylor D. W., Petrera M., Hendry M., Theodoropoulos J. S. (2011). A systematic review of the use of platelet-rich plasma in sports medicine as a new treatment for tendon and ligament injuries. *Clinical Journal of Sport Medicine*.

[6] Kajikawa Y., Morihara T., Sakamoto H. (2008). Platelet-rich plasma enhances the initial mobilization of circulation-derived cells for tendon healing. *Journal of Cellular Physiology*.

[7] McCarrel T. M., Minas T., Fortier L. A. (2012). Optimization of leukocyte concentration in platelet-rich plasma for the treatment of tendinopathy. *The Journal of Bone & Joint Surgery—American Volume*.

[8] Reurink G., Goudswaard G. J., Moen M. H. (2014). Platelet-rich plasma injections in acute muscle injury. *The New England Journal of Medicine*.

[9] Hammond J. W., Hinton R. Y., Curl L. A., Muriel J. M., Lovering R. M. (2009). Use of autologous platelet-rich plasma to treat muscle strain injuries. *The American Journal of Sports Medicine*.

[10] Marx R. E., Carlson E. R., Eichstaedt R. M., Schimmele S. R., Strauss J. E., Georgeff K. R. (1998). Platelet-rich plasma: growth factor enhancement for bone grafts. *Oral Surgery, Oral Medicine, Oral Pathology, Oral Radiology, and Endodontics*.

[11] Weibrich G., Hansen T., Kleis W., Buch R., Hitzler W. E. (2004). Effect of platelet concentration in platelet-rich plasma on peri-implant bone regeneration. *Bone*.

[12] Mazzocca A. D., McCarthy M. B. R., Chowaniec D. M. (2012). The positive effects of different platelet-rich plasma methods on human muscle, bone, and tendon cells. *American Journal of Sports Medicine*.

[13] Schallmoser K., Bartmann C., Rohde E. (2007). Human platelet lysate can replace fetal bovine serum for clinical-scale expansion of functional mesenchymal stromal cells. *Transfusion*.

[14] Anitua E., Sánchez M., Zalduendo M. M. (2009). Fibroblastic response to treatment with different preparations rich in growth factors. *Cell Proliferation*.

[15] Gonzales V. K., de Mulder E. L. W., de Boer T. (2013). Platelet-rich plasma can replace fetal bovine serum in human meniscus cell cultures. *Tissue Engineering—Part C: Methods*.

[16] Foster T. E., Puskas B. L., Mandelbaum B. R., Gerhardt M. B., Rodeo S. A. (2009). Platelet-rich plasma: from basic science to clinical applications. *American Journal of Sports Medicine*.

[17] Pakyari M., Farrokhi A., Maharlooei M. K., Ghahary A. (2013). Critical role of transforming growth factor beta in different phases of wound healing. *Advances in Wound Care*.

[18] Holmes D. I. R., Zachary I. (2005). The vascular endothelial growth factor (VEGF) family: angiogenic factors in health and disease. *Genome Biology*.

[19] Donovan J., Abraham D., Norman J. (2013). Platelet-derived growth factor signaling in mesenchymal cells. *Frontiers in Bioscience*.

[20] Lopez-Vidriero E., Goulding K. A., Simon D. A., Johnson D. H., Sanchez M. (2010). Poor standardization in platelet-rich therapies hampers advancement. *Arthroscopy: The Journal of Arthroscopic and Related Surgery*.

[21] Wright-Carpenter T., Klein P., Schäferhoff P., Appell H. J., Mir L. M., Wehling P. (2004). Treatment of muscle injuries by local administration of autologous conditioned serum: a pilot study on sportsmen with muscle strains. *International Journal of Sports Medicine*.

[22] Mrowiec Z. R., Oleksowicz L., Dutcher J. P., De Leon-Fernandez M., Lalezari P., Puszkin E. G. (1995). A novel technique for preparing improved puffy coat platelet concentrates. *Blood Cells, Molecules, and Diseases*.

[23] Ferizhandy Ali S. (2011). Platelet activation in stored platelet concentrates: comparision of two methods preparation. *Journal of Blood Disorders & Transfusion*.

[24] Bernardini G., Chellini F., Frediani B., Spreafico A., Santucci A. (2015). Human platelet releasates combined with polyglycolic acid scaffold promote chondrocyte differentiation and phenotypic maintenance. *Journal of Biosciences*.

[25] Kon E., Buda R., Filardo G. (2010). Platelet-rich plasma: intra-articular knee injections produced favorable results on degenerative cartilage lesions. *Knee Surgery, Sports Traumatology, Arthroscopy*.

[26] Anitua E., Orive G. (2010). Short implants in maxillae and mandibles: a retrospective study with 1 to 8 years of follow-up. *Journal of Periodontology*.

[27] Anitua E., Pelacho B., Prado R. (2015). Infiltration of plasma rich in growth factors enhances in vivo angiogenesis and improves reperfusion and tissue remodeling after severe hind limb ischemia. *Journal of Controlled Release*.

[28] Lee J.-C., Min H. J., Park H. J., Lee S., Seong S. C., Lee M. C. (2013). Synovial membrane-derived mesenchymal stem cells supported by platelet-rich plasma can repair osteochondral defects in a rabbit model. *Arthroscopy*.

[29] Pihut M., Szuta M., Ferendiuk E., Zeńczak-Więckiewicz D. (2015). Evaluation of pain regression in patients with temporomandibular dysfunction treated by intra-articular platelet-rich plasma injections: a preliminary report. *BioMed Research International*.

[30] Kakudo N., Morimoto N., Kushida S., Ogawa T., Kusumoto K. (2014). Platelet-rich plasma releasate promotes angiogenesis in vitro and in vivo. *Medical Molecular Morphology*.

[31] Lee H.-R., Park K. M., Joung Y. K., Park K. D., Do S. H. (2012). Platelet-rich plasma loaded hydrogel scaffold enhances chondrogenic differentiation and maturation with up-regulation of CB1 and CB2. *Journal of Controlled Release*.

[32] Xie X., Wang Y., Zhao C. (2012). Comparative evaluation of MSCs from bone marrow and adipose tissue seeded in PRP-derived scaffold for cartilage regeneration. *Biomaterials*.

[33] Mishra A., Harmon K., Woodall J., Vieira A. (2012). Sports medicine applications of platelet rich plasma. *Current Pharmaceutical Biotechnology*.

[34] Bausset O., Giraudo L., Veran J. (2012). Formulation and storage of platelet-rich plasma homemade product. *BioResearch Open Access*.

[35] Yamada Y., Ueda M., Naiki T., Takahashi M., Hata K.-I., Nagasaka T. (2004). Autogenous injectable bone for regeneration with mesenchymal stem cells and platelet-rich plasma: tissue-engineered bone regeneration. *Tissue Engineering*.

[36] Hemeda H., Kalz J., Walenda G., Lohmann M., Wagner W. (2013). Heparin concentration is critical for cell culture with human platelet lysate. *Cytotherapy*.

[37] Araki J., Jona M., Eto H. (2012). Optimized preparation method of platelet-concentrated plasma and noncoagulating platelet-derived factor concentrates: maximization of platelet concentration and removal of fibrinogen. *Tissue Engineering—Part C: Methods*.

[38] Bowen R. A. R., Remaley A. T. (2014). Interferences from blood collection tube components on clinical chemistry assays. *Biochemia Medica*.

[39] Amable P. R., Carias R. B. V., Teixeira M. V. T. (2013). Platelet-rich plasma preparation for regenerative medicine: optimization and quantification of cytokines and growth factors. *Stem Cell Research and Therapy*.

[40] Livak K. J., Schmittgen T. D. (2001). Analysis of relative gene expression data using real-time quantitative PCR and the 2^−ΔΔ*C*_T_^ method. *Methods*.

[41] Zhu Y., Yuan M., Meng H. Y. (2013). Basic science and clinical application of platelet-rich plasma for cartilage defects and osteoarthritis: a review. *Osteoarthritis and Cartilage*.

[42] Anitua E., Prado R., Orive G. (2013). Endogenous morphogens and fibrin bioscaffolds for stem cell therapeutics. *Trends in Biotechnology*.

[43] Betsch M., Schneppendahl J., Thuns S. (2013). Bone marrow aspiration concentrate and platelet rich plasma for osteochondral repair in a porcine osteochondral defect model. *PLoS ONE*.

[44] Li H., Usas A., Poddar M. (2013). Platelet-rich plasma promotes the proliferation of human muscle derived progenitor cells and maintains their stemness. *PLoS ONE*.

[45] Castrén E., Sillat T., Oja S. (2015). Osteogenic differentiation of mesenchymal stromal cells in two-dimensional and three-dimensional cultures without animal serum. *Stem Cell Research & Therapy*.

[46] McShine R. L., Das P. C., Smit Sibinga C. T., Brozovic B. (1990). Differences between the effects of EDTA and citrate anticoagulants on platelet count and mean platelet volume. *Clinical and Laboratory Haematology*.

[47] McShine R. L., Das P. C., Sibinga C. T. S., Brozović B. (1991). Effect of EDTA on platelet count and other platelet parameters in blood and blood components collected with CPDA-1. *Vox Sanguinis*.

[48] Araki J., Jona M., Eto H. (2012). Optimized preparation method of platelet-concentrated plasma and noncoagulating platelet-derived factor concentrates: maximization of platelet concentration and removal of fibrinogen. *Tissue Engineering Part C: Methods*.

[49] Ladenson J. H., Tsai L. M. B., Michael J. M., Kessler G., Joist J. H. (1974). Serum versus heparinized plasma for eighteen common chemistry tests: is serum the appropriate specimen?. *American Journal of Clinical Pathology*.

[50] Bath P. M. W., Butterworth R. J. (1996). Platelet size: measurement, physiology and vascular disease. *Blood Coagulation and Fibrinolysis*.

[51] Park Y., Schoene N., Harris W. (2002). Mean platelet volume as an indicator of platelet activation: methodological issues. *Platelets*.

[52] Aster R. H. (2013). Blood platelet kinetics and platelet transfusion. *The Journal of Clinical Investigation*.

[53] Oviedo C., Rodríguez J. (2003). EDTA: the chelating agent under environmental scrutiny. *Quimica Nova*.

[54] Fukaya M., Ito A. (2014). A new economic method for preparing platelet-rich plasma. *Plastic and Reconstructive Surgery—Global Open*.

[55] Lei H., Gui L., Xiao R. (2009). The effect of anticoagulants on the quality and biological efficacy of platelet-rich plasma. *Clinical Biochemistry*.

[56] Pignatelli P., Pulcinelli F. M., Ciatti F. (1995). Acid Citrate Dextrose (ACD) formula A as a new anticoagulant in the measurement of in vitro platelet aggregation. *Journal of Clinical Laboratory Analysis*.

[57] Pignatelli P., Pulcinelli F. M., Ciatti F., Pesciotti M., Ferroni P., Gazzaniga P. P. (1996). Effects of storage on in vitro platelet responses: comparison of ACD and Na citrate anticoagulated samples. *Journal of Clinical Laboratory Analysis*.

[58] Jochems C. E. A., van der Valk J. B. F., Stafleu F. R., Baumans V. (2002). The use of fetal bovine serum: ethical or scientific problem?. *Alternatives to Laboratory Animals*.

[59] Gstraunthaler G. (2003). Alternatives to the use of fetal bovine serum: serum-free cell culture. *ALTEX: Alternativen zu Tierexperimenten*.

[60] Cordeiro-Spinetti E., de Mello W., Trindade L. S., Taub D. D., Taichman R. S., Balduino A. (2014). Human bone marrow mesenchymal progenitors: perspectives on an optimized in vitro manipulation. *Frontiers in Cell and Developmental Biology*.

[61] Eppley B. L., Woodell J. E., Higgins J. (2004). Platelet quantification and growth factor analysis from platelet-rich plasma: implications for wound healing. *Plastic and Reconstructive Surgery*.

[62] Weibrich G., Kleis W. K. G., Hafner G., Hitzler W. E. (2002). Growth factor levels in platelet-rich plasma and correlations with donor age, sex, and platelet count. *Journal of Cranio-Maxillofacial Surgery*.

[63] Sundman E. A., Cole B. J., Fortier L. A. (2011). Growth factor and catabolic cytokine concentrations are influenced by the cellular composition of platelet-rich plasma. *American Journal of Sports Medicine*.

[64] El-Sharkawy H., Kantarci A., Deady J. (2007). Platelet-rich plasma: growth factors and pro- and anti-inflammatory properties. *Journal of Periodontology*.

[65] Castillo T. N., Pouliot M. A., Hyeon Joo Kim, Dragoo J. L. (2011). Comparison of growth factor and platelet concentration from commercial platelet-rich plasma separation systems. *The American Journal of Sports Medicine*.

[66] Rubio-Azpeitia E., Andia I. (2014). Partnership between platelet-rich plasma and mesenchymal stem cells: in vitro experience. *Muscles, Ligaments and Tendons Journal*.

[67] Baksh D., Song L., Tuan R. S. (2004). Adult mesenchymal stem cells: characterization, differentiation, and application in cell and gene therapy. *Journal of Cellular and Molecular Medicine*.

[68] Zhang J., Wang J. H.-C. (2013). Human tendon stem cells better maintain their stemness in hypoxic culture conditions. *PLoS ONE*.

[69] Sung H. J., Hong S. C., Yoo J. H. (2010). Stemness evaluation of mesenchymal stem cells from placentas according to developmental stage: comparison to those from adult bone marrow. *Journal of Korean Medical Science*.

[70] Akeda K., An H. S., Okuma M. (2006). Platelet-rich plasma stimulates porcine articular chondrocyte proliferation and matrix biosynthesis. *Osteoarthritis and Cartilage*.

[71] Spreafico A., Chellini F., Frediani B. (2009). Biochemical investigation of the effects of human platelet releasates on human articular chondrocytes. *Journal of Cellular Biochemistry*.

[72] Gaissmaier C., Fritz J., Krackhardt T., Flesch I., Aicher W. K., Ashammakhi N. (2005). Effect of human platelet supernatant on proliferation and matrix synthesis of human articular chondrocytes in monolayer and three-dimensional alginate cultures. *Biomaterials*.

[73] Kaps C., Loch A., Haisch A. (2002). Human platelet supernatant promotes proliferation but not differentiation of articular chondrocytes. *Medical and Biological Engineering and Computing*.

[74] do Amaral R. J., Matsiko A., Tomazette M. R. (2015). Platelet-rich plasma releasate differently stimulates cellular commitment toward the chondrogenic lineage according to concentration. *Journal of Tissue Engineering*.

